# Longitudinal Diffusion MRI Characterizes Persistent Perivascular Diffusivity Asymmetry and White Matter Abnormalities After Cranioplasty for Decompressive Craniectomy

**DOI:** 10.3390/diagnostics16101502

**Published:** 2026-05-15

**Authors:** Xinyu Xu, Lulu Yang, Jiuyu Gao, Xiaoxuan Li, Yifan Fu, Minghao Xu, Shilin Liu, Yaotian Gao, Keyi Lin, Jifa Xia, Tao Jiang

**Affiliations:** 1 Department of Neurosurgery, The First Affiliated Hospital of Anhui Medical University, Hefei 230022, China; 2445012250@stu.ahmu.edu.cn (X.X.); yfyb12218010@fy.ahmu.edu.cn (L.Y.); 2445012249@stu.ahmu.edu.cn (J.G.); 2345012176@stu.ahmu.edu.cn (X.L.); 2345012177@stu.ahmu.edu.cn (Y.F.); 2345012175@stu.ahmu.edu.cn (M.X.); 2446010267@stu.ahmu.edu.cn (S.L.); gaoyaotian0707@mail.ustc.edu.cn (Y.G.); 2545012349@stu.ahmu.edu.cn (J.X.); 2 Department of Neurosurgery, Anhui Public Health Clinical Center, Hefei 230011, China; linky1119@163.com; 3 Department of Neurosurgery, The Second Affiliated Hospital of Anhui Medical University, Hefei 230601, China

**Keywords:** cranioplasty, DTI-ALPS, postoperative monitoring, white matter microstructure, diffusion MRI

## Abstract

**Background/Objectives:** Delayed neurological deficits after decompressive craniectomy may improve after cranioplasty, but quantitative imaging markers for postoperative monitoring remain limited. This study evaluated diffusion tensor imaging analysis along the perivascular space (DTI-ALPS) as an exploratory diffusion-based marker of defect-referenced perivascular diffusivity asymmetry and examined its relationship with white matter microstructure. **Methods:** Forty-three adults undergoing first-time cranioplasty after decompressive craniectomy and thirty-four matched healthy controls underwent diffusion magnetic resonance imaging and neuropsychological assessment before cranioplasty; twenty-five patients were reassessed at 3 months. DTI-ALPS was quantified globally and according to defect laterality. The white matter microstructure was assessed using tract-based spatial statistics and automated fiber quantification. The associations between imaging measures and cognitive performance were also examined. **Results:** Global DTI-ALPS was significantly lower in patients than in controls both before cranioplasty and at 3-month follow-up, with no significant longitudinal increase. Defect-referenced hemispheric asymmetry persisted, with lower ALPS values on the affected side, and white matter abnormalities were widespread before cranioplasty and remained evident at follow-up. Associations between imaging measures and cognitive performance were not significant after multiple-comparison correction. **Conclusions:** DTI-ALPS may capture persistent defect-related hemispheric diffusion asymmetry after cranioplasty and provide complementary information to conventional white matter metrics. However, in patients with substantial postoperative anatomical and fiber-orientation changes, ALPS should be interpreted cautiously as an exploratory proxy of perivascular diffusivity rather than as a direct measure of glymphatic function or physiological recovery.

## 1. Introduction

Decompressive craniectomy (DC) is a life-saving procedure for refractory intracranial hypertension following severe brain injury, including traumatic brain injury (TBI) and malignant middle cerebral artery infarction [1,2]. By creating a large cranial defect, DC alters cranial boundary conditions and exposes the recovering brain to a prolonged nonphysiological state with abnormal biomechanics and intracranial hydrodynamics prior to reconstruction [3]. Cranioplasty (CP) is therefore performed after stabilization to restore cranial integrity and mechanical protection [2,4]; however, recovery mechanisms, timing, and perioperative monitoring targets remain debated.

During the skull-defect interval, some patients develop delayed neurological deterioration or plateaued recovery in motor, language, or cognitive domains that may partially improve after CP, often described as sinking skin flap syndrome or syndrome of the trephined [3,5]. These observations suggest that CP may influence intracranial physiology beyond cosmetic repair, including cerebrospinal fluid (CSF) hydrodynamics, cerebral perfusion, and intracranial pressure transmission [3,5,6]. However, longitudinal imaging evidence linking cranial defects to brain-wide diffusion abnormalities and functional outcomes remains limited.

The perivascular CSF–interstitial fluid exchange pathway, often described as the “glymphatic” system, may be one mechanism through which altered intracranial dynamics affect recovery after decompressive craniectomy and cranioplasty [7,8]. Previous studies suggest that skull defects can disturb intracranial physiology and that CP may partly improve related abnormalities [9,10]. In humans, direct assessment of this pathway is typically invasive or logistically challenging [11,12]. Diffusion MRI analysis along the perivascular space (DTI-ALPS) provides an indirect diffusion-based proxy related to perivascular diffusivity [13]. However, ALPS should not be regarded as a direct measure of glymphatic flow or clearance, because its estimation depends on local fiber orientation, ROI placement, and periventricular anatomy [13,14]. Therefore, in this study, ALPS was interpreted cautiously as a diffusion-related marker that may reflect both perivascular diffusivity and structural or geometric factors. Within this framework, we examined perioperative hemispheric ALPS patterns and their relationships with white matter microstructure and cognition after cranioplasty.

White matter (WM) microstructure underpins large-scale brain networks, and diffuse WM injury after severe TBI or large-vessel stroke may constrain cognitive recovery. Functional gains are often observed early after CP [5,6], whereas the temporal evolution of WM microstructural abnormalities remains less well characterized. However, whether perioperative diffusion changes and WM recovery follow distinct trajectories remains uncertain and requires cautious longitudinal interpretation. Few studies have jointly tracked perivascular diffusion proxies, WM microstructure, and cognitive outcomes within a unified longitudinal framework.

We performed a longitudinal study of patients undergoing first-time polyetheretherketone (PEEK) cranioplasty after DC and healthy controls. Diffusion MRI was acquired before CP and at postoperative follow-up to quantify global and hemispheric DTI-ALPS indices [13]. We combined tract-based spatial statistics (TBSS) [15] with automated fiber quantification (AFQ) to profile WM microstructure and relate imaging measures to neuropsychological outcomes. Accounting for common indications for DC (TBI and stroke), lesion burden, and laterality, we tested whether ALPS and WM abnormalities are present Pre-CP, change after CP with defect-relative laterality, and relate to defect burden and cognition. This work aimed to characterize perioperative diffusion-related and white matter changes after CP and to explore the value of DTI-ALPS as a candidate marker for postoperative monitoring in a population with complex postoperative anatomy.

## 2. Materials and Methods

### 2.1. Study Design and Participants

**Figure 1:**
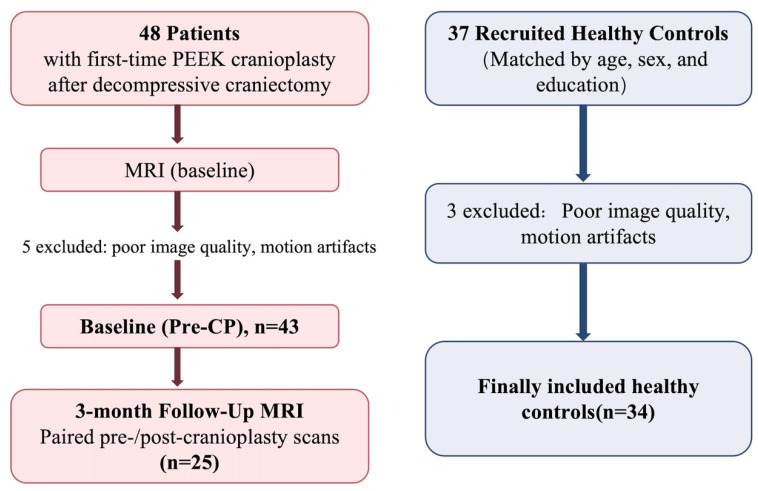
Study design and participant flow.

This prospective study complied with the Declaration of Helsinki (2013 revision), was approved by the Institutional Review Board of The First Affiliated Hospital of Anhui Medical University on 30 May 2025 (IRB No. PJ-YX2025-029), and obtained written informed consent from participants or legal representatives. All participant recruitment and data collection were conducted after ethics approval had been obtained. Baseline pre-cranioplasty assessments were conducted between July 2025 and October 2025, and 3-month postoperative follow-up assessments were completed between October 2025 and January 2026. we consecutively recruited adults (18–65 years) undergoing first-time PEEK cranioplasty (CP) after decompressive craniectomy for traumatic brain injury or malignant middle cerebral artery infarction who were able to complete MRI and neuropsychological testing; detailed inclusion/exclusion criteria are provided in Supplementary Materials. Of 48 enrolled patients, 5 were excluded due to inadequate MRI quality, leaving 43 for baseline analyses; 25 completed the ~3-month (±7 days) follow-up. Reasons for missing follow-up were recorded when available, and baseline characteristics were compared between follow-up completers and non-completers to assess potential attrition-related bias. Healthy controls were community-recruited and matched for age, sex, and education (*n* = 34 after excluding 3 for image quality/motion; eligibility criteria in Supplementary Materials). For hemisphere-specific analyses, the affected hemisphere was defined a priori as ipsilateral to the craniectomy/skull defect/index lesion (contralateral “unaffected”); this definition was applied consistently for ALPS estimation and statistical modeling (Figure 1).

### 2.2. Neuropsychological Assessment

A trained examiner blinded to group and imaging administered tests within 24 h before MRI at each time point. Global cognition was assessed using the Montreal Cognitive Assessment (MoCA) and Mini-Mental State Examination (MMSE); mood symptoms using the Hamilton Depression Rating Scale (HAMD-17) and Hamilton Anxiety Rating Scale (HAMA); and domain-specific performance using the Auditory Verbal Learning Test (AVLT), Trail Making Test Parts A and B, and the Stroop color–word interference test. Higher MoCA/MMSE and Digit Span scores indicate better performance, whereas longer completion times for the Trail Making, Stroop, and AVLT tasks indicate poorer performance.

### 2.3. MRI Acquisition

Diffusion MRI was acquired on a 3.0-T Siemens MAGNETOM Vida scanner (Siemens Healthineers, Erlangen, Germany) using a 64-channel head/neck coil and a single-shot echo-planar imaging (EPI) sequence (64 directions, b = 1000 s/mm^2^, 6 b0; TR/TE = 9000/87 ms; voxel size 0.9 × 0.9 × 3.0 mm^3^). Full MRI protocol and quality-control procedures are provided in Supplementary Materials.

### 2.4. DTI Data Preprocessing and Tensor-Derived Metrics

Preprocessing used MRtrix3 software package (Brain Research Institute, Melbourne, Australia) and FSL (version 5.0.8; FMRIB Software Library, University of Oxford, Oxford, UK) and included Marchenko–Pastur principal component analysis (MP-PCA) denoising [16], Gibbs ringing removal [17], and eddy-current/motion correction using FSL eddy with gradient reorientation [18]. Because reverse phase-encoding images or field maps were unavailable, susceptibility distortion correction was not performed and -rpe_none was used. Potential distortion effects were therefore addressed through visual quality control, exclusion of scans with gross geometric distortion, and cautious interpretation of ALPS estimates. This limitation was considered particularly relevant for ALPS interpretation because the regions of interest (ROIs) were located near the lateral ventricles, where EPI-related geometric distortion may affect directional diffusivity estimates. Diffusion data were visually inspected for motion, signal dropout, and gross geometric distortion; scans with inadequate image quality were excluded from analysis. N4 bias-field correction was performed using ANTs, and a brain mask was generated from b0 images. Tensor fitting in the native diffusion space yielded fractional anisotropy (FA), mean diffusivity (MD), axial diffusivity (AD), radial diffusivity (RD), eigenvalues, and scanner-axis diffusivities (Dxx/Dyy/Dzz) for ALPS estimation.

### 2.5. DTI-ALPS Analysis

**Figure 2:**
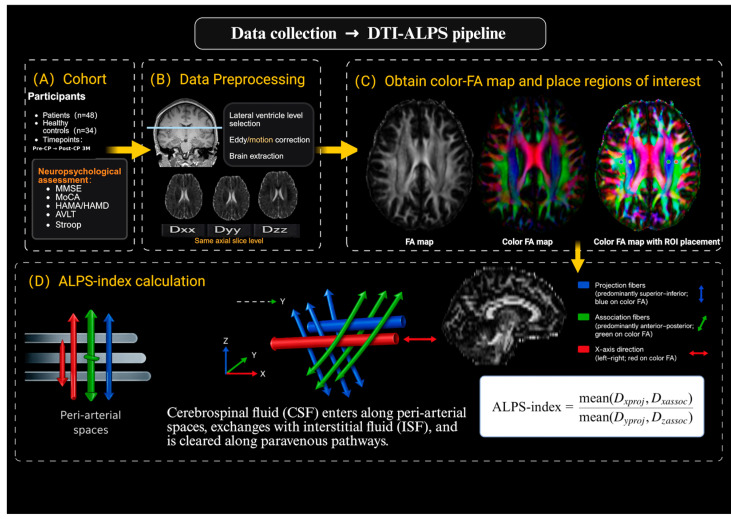
Overview of data collection and DTI-ALPS analysis workflow. (**A**) Cohort composition, follow-up time points, and neuropsychological assessment. (**B**) Diffusion MRI preprocessing and extraction of Dxx, Dyy, and Dzz at the same axial slice level. (**C**) FA/color-FA maps and ROI placement at the lateral ventricular body level. The colored dots indicate representative regions of interest (ROIs) placed in the projection- and association-fiber regions for extracting directional diffusivities used in DTI-ALPS calculation. (**D**) ALPS-index calculation based on directional diffusivities in projection and association fiber regions.

DTI-ALPS was computed following Taoka et al. [13]. On color FA maps at the level of the lateral ventricular body, identical small ROIs were placed in projection and association fiber regions adjacent to the ventricle, avoiding visible CSF partial volume and lesion-related abnormalities. In patients with ventricular deformation or periventricular distortion, ROIs were kept at the same anatomical level and within the expected fiber region whenever possible, while avoiding visibly distorted tissue or lesion-related signal abnormalities. Although this approach helped maintain anatomical consistency across participants and time points, residual effects of altered brain morphology and fiber geometry on ALPS estimation could not be fully excluded. Directional diffusivities were extracted in scanner space. ALPS = (Dx_proj + Dx_assoc)/(Dy_proj + Dz_assoc). Hemisphere-specific ALPS was categorized as affected versus unaffected as defined in Section 2.1; global ALPS was the bilateral mean. Two neuroradiologists blinded to group and outcomes independently placed ROIs and measured ALPS; the mean value was used for analyses [14]. Inter-rater reliability was assessed using a two-way random-effects absolute-agreement intraclass correlation coefficient model and was excellent (ICC = 0.91, 95% CI: 0.85–0.95), supporting the reproducibility of ROI-based ALPS measurements. The DTI-ALPS workflow is summarized in Figure 2.

### 2.6. Tract-Based Spatial Statistics (TBSS) Analysis

TBSS was conducted using FSL (v5.0.8; FMRIB Software Library, University of Oxford, Oxford, UK).. After eddy correction and brain extraction using the Brain Extraction Tool (BET) [19], FA maps were processed with the standard TBSS workflow [15], including nonlinear registration, projection onto a mean FA skeleton (FA > 0.20), and voxel-wise inference. Group comparisons were conducted using FSL randomise (5000 permutations) with threshold-free cluster enhancement (TFCE) and family-wise error (FWE) control at *p*_FWE < 0.05, adjusting for age, sex, and education.

### 2.7. Automated Fiber Quantification (AFQ) Analysis

Automated Fiber Quantification (AFQ toolbox; Stanford University, Stanford, CA, USA; implemented in MATLAB R2024b, The MathWorks, Inc., Natick, MA, USA) was used to characterize tract-specific WM microstructure across 20 major pathways using tract-averaged metrics and 100-node along-tract profiles [20]. Whole-brain deterministic tractography based on the diffusion tensor model was performed [21], followed by standard bundle segmentation and cleaning. Node-wise FA, MD, AD, and RD were extracted; tract-level summaries were computed by averaging across nodes. Tract reconstructions and profiles were visually inspected for quality control.

### 2.8. Statistical Analysis

Statistical analyses were conducted using SPSS (version 26.0; IBM Corp., Armonk, NY, USA) and MATLAB R2024b (The MathWorks, Inc., Natick, MA, USA), with linear mixed-effects models and standard parametric or nonparametric procedures, as appropriate. To accommodate incomplete follow-up, longitudinal changes in global ALPS were assessed using linear mixed-effects models with subject-specific random intercepts and time (Pre-CP, Post-CP 3M) as a fixed effect, with adjustment for age, sex, and education. Hemisphere-specific models additionally included hemisphere (affected vs. contralateral) and a time-by-hemisphere interaction term. For AFQ, node-wise tract profiles were evaluated with covariate adjustment, and multiple comparisons were controlled within each tract using Bonferroni correction across 100 nodes. Tract-mean AFQ comparisons in Supplementary Tables S4–S7 were reported as exploratory analyses using one-way ANOVA with Fisher’s LSD post hoc tests. Imaging–clinical associations were examined using Pearson’s or Spearman’s correlation analyses with covariate adjustment, as appropriate; the prespecified correlation family for ALPS asymmetry was controlled using the Benjamini–Hochberg false discovery rate (FDR) procedure. Given the limited longitudinal sample size, imaging–clinical association analyses were considered exploratory and interpreted cautiously. Etiology was included as an additional covariate in sensitivity analyses, and attrition-related bias was assessed using baseline completer/non-completer comparisons in Supplementary Table S1; subgroup analyses were treated as descriptive due to limited sample size.

## 3. Results

### 3.1. Demographic and Neuropsychological Characteristics

Forty-three patients completed pre-cranioplasty assessment (Pre-CP), and twenty-five returned for the 3-month post-cranioplasty follow-up (Post-CP 3M; paired follow-up); thirty-four matched healthy controls (HCs) were included, with no between-group differences in age, education, or sex distribution (Table 1). At Pre-CP, patients showed lower MMSE and MoCA scores (both q < 0.001), mildly higher HAMD scores (q = 0.019), and poorer performance on Digit Span, Stroop Color, and TMT-A/B (all q ≤ 0.023). For AVLT completion time measures, patients showed longer immediate recall completion time at Pre-CP than HCs (q = 0.013), with no significant difference in delayed recall (q = 0.540) and only a trend for recognition (q = 0.063). At Post-CP 3M, MMSE and MoCA scores remained lower (q = 0.025 and q < 0.001), with persistent deficits in Digit Span backward and TMT-A/B (all q < 0.001), whereas affective symptoms, Stroop Color, Digit Span forward, and AVLT measures did not differ significantly from HCs (all q ≥ 0.090). Completer/non-completer comparisons showed no major systematic baseline differences in most measured variables, although a modest baseline MMSE difference was observed (Supplementary Table S1).

### 3.2. DTI-ALPS Index: Group Differences and Postoperative Longitudinal Changes

**Figure 3:**
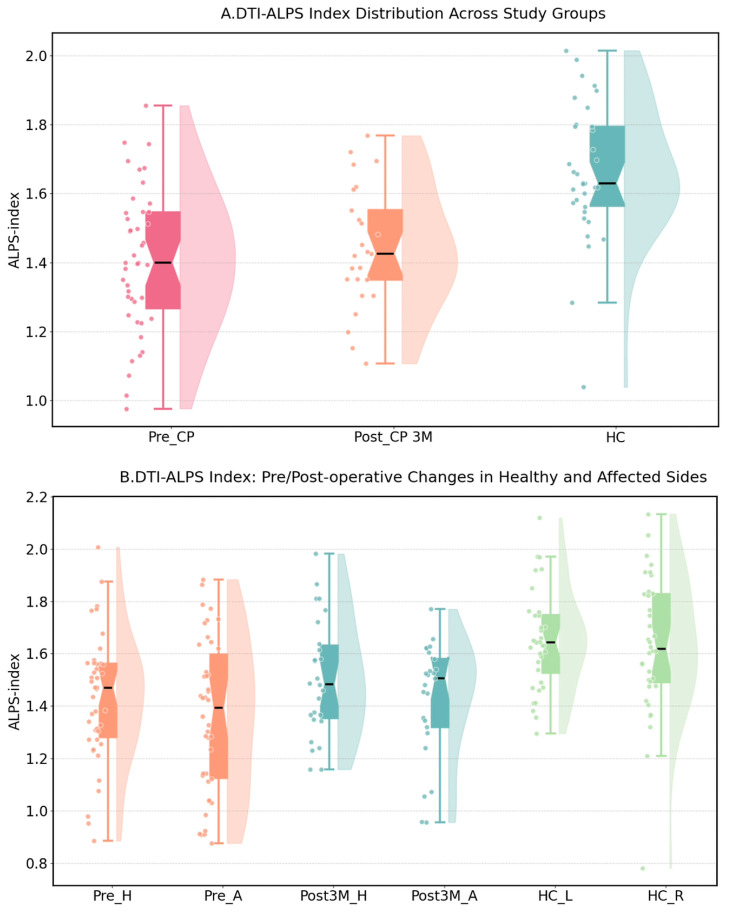
DTI-ALPS at baseline and 3-month follow-up, and hemispheric asymmetry. (**A**) Global DTI-ALPS in the Pre-CP (*n* = 43), Post-CP 3M (*n* = 25), and healthy control (HC, *n* = 34) groups. (**B**) Hemisphere-specific DTI-ALPS on the contralateral/healthy (H) and affected/defect (A) sides at each time point, with HCs shown as the reference. Dots represent individual participants; shaded areas indicate data distributions; black line elements indicate the mean ± standard deviation. Comparisons were performed using linear mixed-effects models.

Global DTI-ALPS was lower in patients at Pre-CP (1.405 ± 0.215, *n* = 43) than in healthy controls (HCs; 1.640 ± 0.204, *n* = 34; *p* < 0.0001). At Post-CP 3M, global DTI-ALPS remained lower than in HCs (1.442 ± 0.214, *n* = 25; *p* < 0.0001) and largely overlapped the lower range of the HC distribution, while the Pre-CP–Post-CP 3M global difference was not significant (Supplementary Table S2; Figure 3). Hemisphere-specific values were approximately symmetric in HCs (HC_L: 1.650 ± 0.187; HC_R: 1.632 ± 0.263), whereas patients tended to show lower ALPS on the affected/defect side than on the contralateral side at both Pre-CP (1.360 ± 0.293 vs. 1.430 ± 0.240) and Post-CP 3M (1.424 ± 0.222 vs. 1.506 ± 0.224) (Supplementary Table S2; Figure 3). These results indicate persistent ALPS-related hemispheric asymmetry after cranioplasty, while global ALPS did not show a significant postoperative increase. In etiology-adjusted sensitivity models, the main ALPS pattern remained unchanged: global ALPS remained lower in patients than in HCs at both Pre-CP and Post-CP 3M, whereas the longitudinal Pre-CP to Post-CP 3M change remained non-significant. Inter-rater agreement for ALPS measurements was excellent (ICC = 0.91, 95% CI: 0.85–0.95).

### 3.3. Tract-Based Spatial Statistics (TBSS): Whole-Brain White Matter Alterations

**Figure 4:**
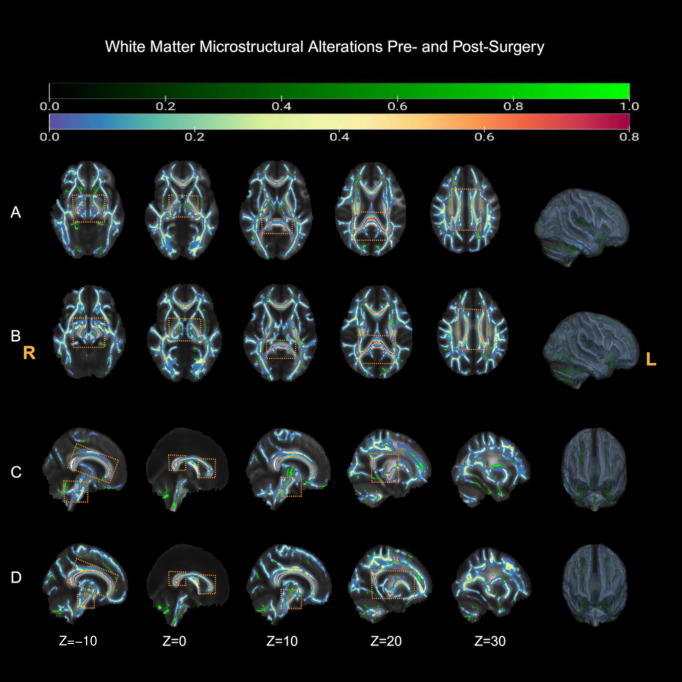
TBSS FA differences between patients and healthy controls. (**A**) Pre-CP vs. HC, axial views. (**B**) Post-CP 3M vs. HC, axial views. (**C**) Pre-CP vs. HC, sagittal views. (**D**) Post-CP 3M vs. HC, sagittal views. Significant FA reductions in patients are shown on the mean FA skeleton in MNI152 space after threshold-free cluster enhancement with family-wise error correction (TFCE-FWE, *p*_FWE < 0.05). Green overlays indicate the mean FA skeleton; blue-to-red overlays indicate significant FA reduction clusters, with warmer colors indicating stronger statistical evidence. Orange dashed boxes highlight representative prominent clusters to improve visual readability; R/L denotes right/left.

With threshold-free cluster enhancement (TFCE) and family-wise error (FWE) correction (*p* < 0.05), TBSS identified widespread FA abnormalities in patients relative to healthy controls at both Pre-CP and Post-CP 3M (Figure 4; Supplementary Table S3). The Pre-CP vs. HC contrast was dominated by a large cluster (86,390 voxels; TFCE-corrp_max = 1.00; peak MNI −8, −51, −50), accompanied by two small clusters (30 and 16 voxels; TFCE-corrp_max = 0.99; peaks −14, −65, −31 and −11, −63, −27). The Post-CP 3M vs. HC contrast similarly revealed an extensive large cluster (82,317 voxels; TFCE-corrp_max = 1.00; peak 2, −41, −56). No within-patient FA clusters survived correction for the longitudinal comparison (Pre-CP vs. Post-CP 3M); therefore, these TBSS results are interpreted as cross-sectional patient–control differences at each time point rather than as evidence of postoperative FA change.

### 3.4. AFQ-Based Tract-Specific Analysis

**Figure 5:**
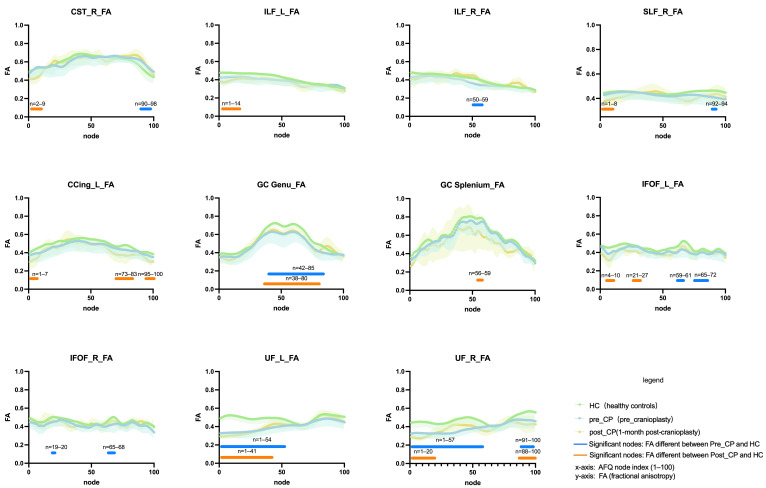
Node-wise FA profiles along selected white matter tracts. FA across 100 nodes per tract in HC, Pre-CP, and Post-CP 3M (paired *n* = 25). Horizontal bars indicate significant node segments after within-tract Bonferroni correction (α = 0.05/100).

Across 20 tracts (Supplementary Tables S4–S7; Figure 5), AFQ localized patient–control differences to major commissural, association, and projection pathways. At Pre-CP, FA was reduced in 12/20 tracts, accompanied by increased MD (9/20) and RD (12/20), with limited AD changes (ATR_L and SLF_L) (post hoc *p* < 0.05; Supplementary Tables S4–S7). At Post-CP 3M, FA remained lower in 5/20 tracts (callosal splenium, left CCing/ILF, right SLF/UF), while callosal genu and left IFOF showed trend-level differences (0.05 < *p* < 0.10). MD and RD remained elevated in 8/20 tracts in post hoc groupwise comparisons (*p* < 0.05). AD showed bidirectional changes, with increases in CST_L and SLF_L and decreases in callosal splenium and AF_R in post hoc comparisons (*p* < 0.05). Full tract names and abbreviations are provided in the notes for Supplementary Tables S4–S7. In paired longitudinal analyses (Pre-CP vs. Post-CP 3M), tract-mean FA and RD were unchanged, whereas MD increased in CST_R, ILF_L, and AF_R and AD increased in CST_L (*p* < 0.05).

### 3.5. Node-Wise Tract Profile Analysis

Node-wise analyses (100 nodes per tract; within-tract Bonferroni correction, α = 0.05/100) identified significant contiguous node segments at both Pre-CP and Post-CP 3M (*n* = 25) (Figure 5). At Pre-CP, FA differences were observed in the distal right CST (nodes 90–98), the mid right ILF (50–59), a focal segment of the right SLF (92–94), an extended segment of the callosal genu (42–85), bilateral UF (UF_L 1–54; UF_R 1–57 and 91–100), the callosal splenium (node 3), and bilateral IFOF. At Post-CP 3M, FA differences were observed in the left cingulum (cingulate portion; CCing; 1–7, 73–83, 95–100), the anterior left ILF (1–14), and the anterior left IFOF (4–10, 21–27). At Post-CP 3M, MD and RD differences involved large UF segments (UF_R 1–100; UF_L 1–73) and long ILF segments (ILF_L 16–97; ILF_R 73–100); RD differences also involved ILF_L segments 3–18 and 26–97. Additional segments were detected in the CST, SLF, callosal genu/splenium, and IFOF. AD effects were more spatially restricted, primarily involving the CST, ILF, callosal genu/splenium, and UF. Collectively, the node-wise results highlight spatially localized alterations along tract trajectories, complementing the tract-mean metrics.

### 3.6. Correlations of the ALPS Index with Clinical and White Matter Metrics

**Figure 6:**
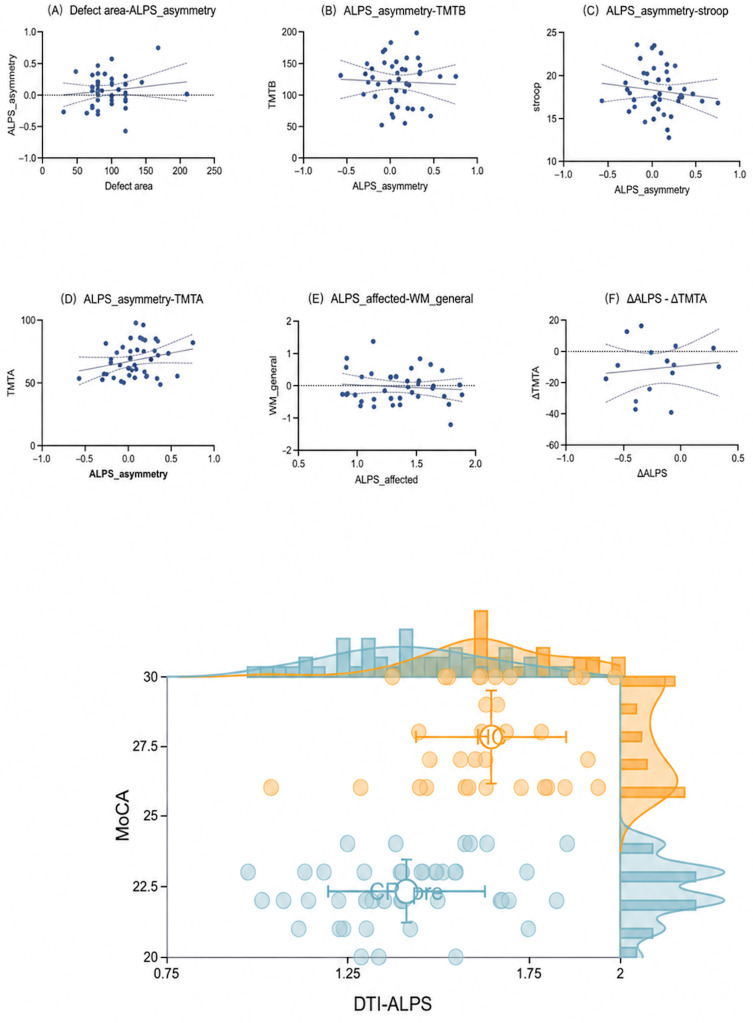
Associations with ALPS metrics. (**A**) Defect area versus ALPS asymmetry. (**B**–**D**) ALPS asymmetry versus executive measures (TMT-B, Stroop, TMT-A). (**E**) ALPS_affected versus WM_general. (**F**) Exploratory association between ΔALPS (post–pre) and ΔTMT-A (post–pre). The two unlabeled scatterplots show DTI-ALPS versus MoCA in HCs (*n* = 34) and the Pre-CP cohort (*n* = 43), respectively. Solid lines indicate least-squares regression fits, dashed lines indicate 95% confidence intervals, and horizontal dotted lines indicate zero/reference lines where applicable. Prespecified tests were two-tailed and FDR-corrected.

After covariate adjustment and FDR correction across prespecified tests, ALPS asymmetry showed no significant associations with defect area or executive measures. DTI-ALPS exhibited only weak, non-significant positive trends with MoCA in both patients and controls (Figure 6). The exploratory analyses did not reveal significant associations between the ALPS metrics and global WM FA integrity or longitudinal changes in executive performance. Taken together, these analyses did not identify robust associations after correction; however, these null findings should be interpreted cautiously given the limited statistical power, particularly for longitudinal change and imaging–cognition analyses.

## 4. Discussion

This study used longitudinal diffusion MRI to characterize perioperative ALPS-related diffusivity and white matter microstructural changes in patients undergoing cranioplasty after decompressive craniectomy. By combining DTI-ALPS with TBSS and AFQ, we found that patients showed reduced ALPS during the skull-defect stage, persistent defect-referenced hemispheric asymmetry after cranioplasty, and widespread white matter abnormalities that remained evident at 3 months. These combined findings suggest that ALPS may provide complementary information on perioperative diffusion patterns beyond conventional white matter metrics. At the same time, because ALPS is an indirect diffusion-based measure and depends on local periventricular anatomy and fiber orientation, the observed changes should be interpreted as ALPS-related diffusion alterations rather than direct evidence of glymphatic recovery or postoperative physiological restoration. Accordingly, the ALPS findings were interpreted as diffusion-related periventricular/perivascular-region observations, not as direct evidence of glymphatic flow or clearance.

The preoperative reduction in DTI-ALPS is compatible with DC-related disruption of intracranial boundary conditions and partially reversible dysfunction (sinking skin flap syndrome/syndrome of the trephined, SoT). Larger defects may alter CSF dynamics, attenuate intracranial pressure fluctuation transmission, and perturb perfusion, thereby affecting periventricular diffusion patterns and perivascular water mobility. To better capture defect-related vulnerability, hemispheric indices were uniformly referenced to the defect side; etiology was included as a covariate to partially address heterogeneity.

In the paired 3-month follow-up subsample, global DTI-ALPS did not show a significant longitudinal increase, whereas defect-referenced hemispheric asymmetry remained evident. This pattern suggests that side-specific ALPS information may capture perioperative diffusion features that are not fully represented by global averages. Such hemispheric differences may relate to postoperative changes in water content, vascular pulsatility, perfusion regulation, or intracranial dynamics after reconstruction. At the same time, they may also be influenced by partial geometric normalization, ROI localization, and changes in local fiber orientation. Therefore, the hemispheric ALPS findings are best interpreted as diffusion-related observations that complement conventional white matter metrics, rather than as direct evidence of hemispheric physiological recovery.

Longitudinal interpretation should also consider the different imaging conditions before and after cranioplasty, with a skull defect before surgery and PEEK reconstruction at follow-up. Although PEEK is generally MRI-compatible, diffusion-weighted EPI remains sensitive to susceptibility-related distortion and signal heterogeneity, which may partly influence postoperative ALPS measurements.

Under stringent multiple-comparison correction, TBSS and AFQ consistently indicated persistent WM abnormalities evident at the postoperative follow-up, with AFQ providing tract-level anatomical localization. These findings were interpreted as persistent WM injury burden rather than reversible perioperative change. The absence of robust short-term longitudinal changes suggests that WM microstructural abnormalities may persist during early follow-up, but it should not be interpreted as definitive evidence of a distinct structural recovery trajectory. Longer observation and larger cohorts are needed to characterize temporal evolution more precisely.

TBSS–AFQ integration provided complementary information: TBSS was sensitive to spatially widespread between-group effects, whereas AFQ improved anatomical interpretability and localization of segmental abnormalities potentially diluted in skeleton-based voxel-wise analyses. Convergent evidence supports persistent early postoperative white matter abnormalities.

From a clinical perspective, DTI-ALPS may provide complementary information for describing perioperative hemispheric diffusion patterns after CP, especially when interpreted together with white matter metrics and cognitive measures. In this cohort, however, ALPS was not significantly associated with cognitive performance or global white matter integrity after correction. Therefore, its clinical role should remain exploratory. The present findings do not support the use of DTI-ALPS as a stand-alone tool for clinical decision-making or prognostic stratification, but they support further testing of whether ALPS adds value to conventional imaging and clinical variables in larger longitudinal cohorts. Cognitive deficits mainly involved global cognition and executive/processing-speed domains, broadly paralleling persistent diffusion and WM abnormalities, although corrected imaging–cognition associations were not robust.

Limitations/interpretation. DTI-ALPS is an indirect diffusion-based proxy rather than a direct measure of glymphatic function or physiological recovery. In this post-DC/CP cohort, brain displacement, ventricular deformation, altered CSF distribution, lesion-related white matter abnormalities, and changes in fiber orientation may affect ROI localization and ALPS estimation. Although ROIs were placed carefully to avoid visible CSF partial volume and distorted or lesioned tissue, subtle residual effects of altered brain morphology and fiber geometry may remain. Residual EPI-related distortion remains an important limitation, especially for ALPS ROIs near the lateral ventricles. Because reverse phase-encoding images or field maps were unavailable, formal susceptibility distortion correction could not be performed; therefore, the ALPS findings were interpreted cautiously as diffusion-related observations rather than definitive biological changes in perivascular flow. Follow-up attrition may introduce selection bias; therefore, completer/non-completer baseline comparisons were added, and longitudinal findings were interpreted cautiously as observations from the retained follow-up cohort. The study may also have been underpowered to detect modest longitudinal effects or imaging–cognition associations; therefore, non-significant findings should not be interpreted as evidence of no relationship. Mixed etiology may still influence TBSS/AFQ and ALPS findings; because etiology-specific longitudinal analyses were underpowered, these trajectories should be examined in larger cohorts with sufficient subgroup power. Finally, perioperative modifiers (e.g., sleep, hydration, medication, circadian timing) were not systematically controlled. A longer prospective follow-up with standardized or automated ROI protocols, test–retest reliability assessment, and multimodal physiologic measures will be needed to clarify the specificity of ALPS-related diffusion patterns and their relationship to longer-term outcomes.

## 5. Conclusions

In this prospective longitudinal cohort, DTI-ALPS was reduced during the skull-defect stage and defect-referenced hemispheric asymmetry persisted after cranioplasty, while white matter abnormalities remained evident at early postoperative follow-up. These findings suggest that ALPS may provide complementary diffusion-related information after cranioplasty but should be interpreted cautiously as an exploratory proxy of perivascular diffusivity rather than a direct marker of glymphatic recovery or distinct recovery trajectories. Larger longitudinal studies with standardized ALPS protocols and multimodal physiological measures are needed to clarify its specificity and clinical value.

## Figures and Tables

**Table 1:** Demographic and neuropsychological profiles of patients undergoing cranioplasty and healthy controls.

Variable	Pre-CP (*n* = 43)	Post-CP 3M (*n* = 25)	HC (*n* = 34)	t	*p*/q	t	*p*/q
				(Pre vs. HC)		(Post vs. HC)	
Age(years)	48.10 ± 14.0	47.2 ± 11.80	50.4 ± 6.17	0.89	0.375	1.82	0.076
Education(years)	7.24 ± 2.12	6.98 ± 1.58	8.42 ± 4.36	1.56	0.124	1.04	0.303
Sex (Male/Female)	25/18 (58.14/41.86)	15/10 (60.00/40.00)	16/18 (47.06/52.94)	-	0.366	-	0.430
**Cognitive Assessment**							
MMSE	27.23 ± 2.02	28.56 ± 1.42	29.12 ± 1.23	4.79	<0.001	2.32	0.025
MoCA	20.83 ± 2.30	23.80 ± 2.71	27.83 ± 2.50	12.76	<0.001	9.77	<0.001
HAMD	4.23 ± 3.81	3.21 ± 2.81	2.52 ± 1.88	2.39	0.019	1.33	0.189
HAMA	3.99 ± 3.53	3.41 ± 3.16	3.42 ± 1.73	0.86	0.391	0.06	0.951
**Attention/Concentration**							
WAIS Digit Span (forward)	6.71 ± 2.52	7.38 ± 2.14	7.81 ± 1.25	2.32	0.023	1.73	0.090
WAIS Digit Span(backward)	4.58 ± 1.71	5.74 ± 1.52	6.70 ± 1.32	5.95	<0.001	3.63	<0.001
Stroop Color Test	18.23 ± 2.58	16.59 ± 2.98	15.67 ± 3.67	3.59	<0.001	1.72	0.091
Trail Making A	68.27 ± 13.21	47.46 ± 12.36	36.43 ± 10.52	11.47	<0.001	6.74	<0.001
**Memory (AVLT)**							
Immediate Recall	13.08 ± 2.58	12.33 ± 2.08	11.58 ± 2.54	2.55	0.013	1.33	0.192
Delayed Recall	13.10 ± 2.60	12.95 ± 2.75	12.70 ± 2.50	0.62	0.540	0.36	0.720
Recognition	11.23 ± 3.16	12.47 ± 2.87	12.43 ± 2.19	1.88	0.063	0.69	0.493
**Information Processing and Executive Function**							
Trail Making B	120.82 ± 35.26	102.46 ± 30.26	85.46 ± 18.98	5.27	<0.001	4.78	<0.001
Stroop Word Test	27.34 ± 4.78	22.29 ± 3.89	20.01 ± 5.86	6.05	<0.001	1.32	0.195
Stroop Interference Test	35.79 ± 11.21	34.78 ± 10.03	30.18 ± 6.87	2.56	0.013	1.55	0.129

Note. Values are mean ± standard deviation (SD). Patients completed Pre-CP (*n* = 43); 25 returned at 3 months (Post-CP 3M), providing paired follow-up data. Comparisons with healthy controls (HC, *n* = 34) used two-sided independent-samples t tests for continuous variables and Fisher’s exact tests for sex. Higher scores indicate better performance for MMSE, MoCA, and Digit Span; longer times indicate worse performance for Stroop, Trail Making, and AVLT (seconds). *p* values for demographic comparisons are unadjusted, whereas q values for neuropsychological measures are FDR-adjusted. Longitudinal paired results are reported in the main analyses.

## Data Availability

The data presented in this study are available from the corresponding author upon reasonable request. The data are not publicly available due to privacy and ethical restrictions, as they contain potentially identifiable clinical, neuropsychological, and neuroimaging information from human participants. Access to de-identified data may be considered upon reasonable request and subject to appropriate institutional approval.

## Supplementary Materials

The following supporting information can be downloaded at https://www.mdpi.com/article/10.3390/diagnostics16101502/s1, Methods S1. Eligibility criteria. Methods S2. MRI Acquisition. Methods S3. Statistical analysis. Results S1. Imaging–clinical correlations. Table S1. Baseline characteristics of patients with and without 3-month postoperative follow-up. Table S2. DTI-ALPS index (global and hemispher e-specific) and betweengroup comparisons. Table S3. Significant FA clusters identified by TBSS. Table S4. Group comparisons of tract-mean fractional anisotropy (FA) in 20 major white-matter tracts derived from automated fiber quantification (AFQ). Table S5. Group comparisons of tract-mean mean diffusivity (MD) in 20 major white-matter tracts derived from automated fiber quantification (AFQ). Table S6. Group comparisons of tract-mean axial diffusivity (AD) in 20 major white-matter tracts derived from automated fiber quantification (AFQ). Table S7. Group comparisons of tract-mean radial diffusivity (RD) in 20 major white-matter tracts derived from automated fiber quantification (AFQ).
