# Supplementary material for: Longitudinal Diffusion MRI Characterizes Persistent Perivascular Diffusivity Asymmetry and White Matter Abnormalities After Cranioplasty for Decompressive Craniectomy
Source: Diagnostics (Basel). 2026 May 15;16(10):1502. doi: 10.3390/diagnostics16101502 (PMC13205448; doi:10.3390/diagnostics16101502)
Supplement: Supplementary file 1 [file diagnostics-16-01502-s001.zip › diagnostics-4214922-supplementary.pdf]

Supplementary Data

Supplementary Methods S1. Eligibility criteria

Patients—Inclusion criteria

Patients were eligible if they met all the following:

1. traumatic brain injury (TBI) or malignant middle cerebral artery infarction (MCA infarction) with a skull defect requiring cranioplasty.
2. age 18–65 years.
3. first-time polyetheretherketone (PEEK) cranioplasty.
4. no major systemic disease and no neurological disorders unrelated to the index lesion.
5. no pre-event subjective cognitive complaints.
6. ability to complete MRI and the neuropsychological assessment battery.

Patients—Exclusion criteria

Patients were excluded for any of the following:

1. major neurological disease unrelated to the index lesion.
2. other neurological or psychiatric disorders.
3. surgical contraindications or severe postoperative complications (e.g., intracranial infection, subcutaneous effusion, implant rejection).
4. contraindications to MRI.
5. severe cardiac, hepatic, or renal dysfunction, or alcohol/substance dependence.
6. MRI data of insufficient quality for reliable analysis (e.g., excessive head motion or marked susceptibility artifacts).

Healthy controls—Inclusion criteria

Healthy controls (HCs) were eligible if they met all of the following:

1. age ≥18 years.
2. right-handedness.
3. ability to complete MRI and neuropsychological testing independently.

Healthy controls—Exclusion criteria

HCs were excluded for any of the following.

1. Hamilton Depression Rating Scale–17 item (HAMD-17) score >7.
2. current or lifetime psychiatric diagnosis.
3. serious systemic disease (including severe cardiovascular or cerebrovascular disorders).
4. excessive head motion or poor diffusion MRI quality precluding reliable analysis.

Supplementary Methods S2. MRI Acquisition

All MRI data were acquired on a 3.0-T Siemens MAGNETOM Vida scanner (Siemens Healthineers, Erlangen, Germany) using a 64-channel head/neck coil. High-resolution T1-weighted images were obtained using a 3D MPRAGE sequence (TR/TE/TI = 2300/2.26/1200 ms; flip angle = 7°; matrix = 256 × 256; 1-mm isotropic resolution; sagittal acquisition). Diffusion MRI was acquired using a spin-echo single-shot EPI sequence with GRAPPA (acceleration factor = 2) (TR/TE = 9000/87 ms; matrix = 128 × 128; voxel size = 0.9 × 0.9 × 3.0 mm^3; 64 diffusion-encoding directions at b = 1000 s/mm^2 and 6 b = 0 images; total 70 volumes). The phase-encoding direction was j− and the total readout time was 0.0854 s (recorded for preprocessing; see Section 2.4). All T1-weighted and diffusion images underwent visual quality

control for motion and susceptibility-related artifacts; scans failing predefined criteria were excluded. Resting-state BOLD fMRI was acquired but is not reported in the present study.

Supplementary Methods S3. Statistical analysis

Statistical analyses were conducted using SPSS (version 26.0) and MATLAB. Continuous variables were analyzed using parametric or nonparametric tests, as appropriate, and categorical variables were compared using χ^2 tests or Fisher's exact tests. Demographic comparisons were reported without correction, whereas neuropsychological comparisons were interpreted using FDR-adjusted q values as reported in the main Table 1. Longitudinal changes in global ALPS were evaluated in paired participants (n=25) using linear mixed-effects models with subject-specific random intercepts and time (Pre-CP, Post-CP 3M) as a fixed effect, with adjustment for age, sex, and education. Hemisphere-specific models additionally included hemisphere (affected vs contralateral/unaffected) and a time-by-hemisphere interaction. For AFQ tract profiles, node-wise effects were tested using node-wise linear models with covariate adjustment, and multiplicity was controlled within each tract using Bonferroni correction across 100 nodes (α=0.05/100). Imaging–clinical associations were assessed using Pearson or Spearman correlation analyses with covariate adjustment, as appropriate; ALPS asymmetry was defined as (ALPS_unaffected − ALPS_affected)/(ALPS_unaffected + ALPS_affected), where positive values indicate higher contralateral ALPS, and WM_general was defined as the mean tract-mean FA across AFQ tracts. For the primary correlation family, Benjamini–Hochberg false discovery rate correction was applied across four prespecified tests (defect area, TMT-A, TMT-B, and Stroop measures versus ALPS_asym), whereas all other correlations were considered exploratory.

Supplementary Results S1. Imaging–clinical correlations

With covariate-adjusted partial correlations and FDR correction across four prespecified tests (Fig. 6), ALPS asymmetry was not associated with defect area ($r = 0.138$, 95% CI −0.169 to 0.421, $p = 0.377$; $n = 43$) and showed no robust associations with executive measures (TMT-A $r = 0.257$, $p = 0.096$; TMT-B $r = -0.043$, $p = 0.786$; Stroop $r = -0.132$, $p = 0.399$); none survived FDR correction ($q_{FDR} > 0.05$). DTI-ALPS showed weak, non-significant positive associations with MoCA in both cohorts (Pre-CP $r = 0.202$, $p = 0.194$, $n = 43$; healthy controls $r = 0.195$, $p = 0.268$, $n = 34$). Exploratory analyses further indicated that ALPS_affected was not associated with WM_general ($r = -0.105$, 95% CI −0.411 to 0.222, $p = 0.531$; $n = 43$), and ΔALPS was not associated with ΔTMT-A in the longitudinal subset ($r = 0.117$, 95% CI −0.420 to 0.594, $p = 0.678$; $n = 25$). Overall, no robust linear associations were observed between ALPS metrics and defect burden, global WM FA integrity, or executive performance.

Supplementary Table S1. Baseline characteristics of patients with and without 3-month postoperative follow-up.

Variable	Completed Follow-up (n=25)	Not Completed (n=18)	P value
Age (years)	47.20 ± 11.80	49.35 ± 16.88	0.646
Sex (male), n (%)	15 (60.0%)	10 (55.6%)	1.000
Education (years)	6.98 ± 1.58	7.60 ± 2.71	0.392
Etiology (TBI/MCAI), n	11/14	9/9	0.763
MoCA score	21.13 ± 1.60	20.41 ± 3.02	0.368
MMSE score	27.83 ± 1.80	26.40 ± 2.06	0.024
Global ALPS index	1.38 ± 0.24	1.44 ± 0.18	0.351

Note. Values are presented as mean ± SD or n (%), as appropriate. P values were obtained using independent-samples t tests, Mann–Whitney U tests, chi-square tests, or Fisher’s exact tests according to variable type and distribution. These analyses were performed to assess potential attrition-related bias. Abbreviations: TBI, traumatic brain injury; MCAI, malignant middle cerebral artery infarction; MoCA, Montreal Cognitive Assessment; MMSE, Mini-Mental State Examination; ALPS, analysis along the perivascular space.

Supplementary Table S2. DTI-ALPS index (global and hemisphere-specific) and between-group comparisons.

Group	n	Global DTI-ALPS index (Mean ± SD)	vs HC p (R^2)	vs Pre-CP p (R^2)	Hemisphere-specific Affected/Left (Mean ± SD)	Hemisphere-specific Contralateral/Right (Mean ± SD)
HC	34	1.640±0.204	—	—	1.650±0.187	1.632±0.263
Pre-CP	43	1.405±0.215	<0.0001 (0.238)	—	1.360±0.293	1.430±0.240
Post-CP 3M	25	1.442±0.214	<0.0001 (0.431)	0.495 (0.007)	1.424±0.222	1.506±0.224

Note. Data are presented as mean ± SD. Patient-control comparisons were assessed using covariate-adjusted linear regression, whereas longitudinal Pre-CP versus Post-CP 3M comparisons were assessed using linear mixed-effects models with subject-specific random intercepts. “Affected/Left” and “Contralateral/Right” indicate hemisphere-specific ALPS values (left/right hemispheres in HCs). Abbreviations: ALPS, analysis along the perivascular space; DTI, diffusion tensor imaging; HC, healthy controls; Pre-CP, pre-cranioplasty; Post-CP 3M, 3 months post-cranioplasty. Significance was set at $p < 0.05$.

Supplementary Table S3. Significant FA clusters identified by TBSS.

Contrast	Cluster index	Cluster size (voxels)	Peak TFCE-corrp (MAX)	Peak MNI coordinates (x, y, z, mm)
Pre-CP vs HC	3	86,390	1.00	(-8, -51, -50)
	2	30	0.99	(-14, -65, -31)
	1	16	0.99	(-11, -63, -27)
Post-CP 3M vs HC	1	82,317	1.00	(2, -41, -56)

Note: TBSS was performed on FA maps. Voxel-wise comparisons (Pre-CP vs HC; Post-CP 3M vs HC) were tested using TFCE with FWE correction (voxel-wise $p < 0.05$). Cluster size indicates the number of suprathreshold voxels on the TBSS skeleton. TFCE-corrp_max is the maximum value from the TFCE–FWE corrected probability (corrp) map output by FSL randomise (values closer to 1 indicate stronger evidence; approximately $1 - p_{FWE}$). No clusters with higher FA in patients than in controls were detected.

Supplementary Table S4. Group comparisons of tract-mean fractional anisotropy (FA) in 20 major white-matter tracts derived from automated fiber quantification (AFQ).

Tract	n_pre:n_post:n_HC	Pre-CP (N=43)	Post-CP (N=25)	HC (N=34)	ANOVA		Post-hoc groupwise (LSD)		
					F	p	Pre vs post	Pre vs HC	Post vs HC
ATR_L	39:24:34	0.430±0.058	0.461±0.044	0.441±0.030	1.538	0.222	0.089	0.373	0.258
ATR_R	38:23:30	0.437±0.062	0.424±0.058	0.428±0.050	0.180	0.836	0.789	0.559	0.944
CST_L	38:25:34	0.592±0.070	0.603±0.063	0.623±0.031	3.143	0.049*	0.301	0.015*	0.238
CST_R	39:24:28	0.604±0.065	0.568±0.085	0.601±0.039	3.078	0.051	0.117	0.857	0.155
CCing_L	38:23:32	0.446±0.075	0.436±0.061	0.490±0.049	4.655	0.013*	0.707	0.008**	0.036*
CCing_R	38:22:29	0.412±0.053	0.460±0.034	0.447±0.051	2.820	0.071	0.147	0.039*	0.703
CHippo_L	23:14:9	0.376±0.064	0.374±0.031	0.396±0.037	0.457	0.637	0.952	0.364	0.524
CHippo_R	21:14:10	0.361±0.038	0.352±0.010	0.380±0.025	0.836	0.445	0.796	0.230	0.470
CC Splenium	38:21:32	0.559±0.069	0.506±0.080	0.591±0.034	5.253	0.008**	0.085	0.0455*	0.006**
CC Genu	35:21:34	0.483±0.082	0.491±0.066	0.534±0.026	6.106	0.004**	0.735	0.001**	0.084
IFOF_L	39:22:34	0.416±0.045	0.422±0.048	0.451±0.032	5.475	0.006**	0.708	0.002**	0.068
IFOF_R	40:22:34	0.422±0.041	0.428±0.030	0.454±0.031	6.157	0.004**	0.749	0.001**	0.114
ILF_L	39:23:34	0.385±0.037	0.385±0.046	0.413±0.033	4.991	0.009**	0.988	0.004**	0.043*
ILF_R	35:23:34	0.375±0.038	0.381±0.046	0.400±0.029	4.163	0.019*	0.652	0.005**	0.173
SLF_L	37:23:33	0.402±0.055	0.424±0.052	0.424±0.035	1.856	0.164	0.206	0.077	0.964
SLF_R	38:22:33	0.428±0.043	0.406±0.055	0.450±0.036	4.480	0.015*	0.173	0.043*	0.008**
UF_L	39:22:34	0.399±0.048	0.413±0.059	0.419±0.036	1.401	0.254	0.415	0.101	0.744
UF_R	36:22:34	0.387±0.042	0.378±0.043	0.414±0.029	5.942	0.004**	0.512	0.004**	0.012*
AF_L	36:22:34	0.465±0.051	0.483±0.050	0.490±0.038	2.063	0.135	0.334	0.048*	0.694
AF_R	35:22:32	0.448±0.053	0.447±0.049	0.470±0.038	1.706	0.190	0.935	0.094	0.244

Note: Values are mean ± SD. n_pre:n_post:n_HC indicates the number of participants with valid tract reconstructions in the Pre-CP, Post-CP, and healthy control groups. One-way ANOVA across groups with Fisher's LSD post-hoc tests. *, **, and *** denote $p < 0.05$, $p < 0.01$, and $p < 0.001$, respectively. **Abbreviations:** ATR, anterior thalamic radiation; CST, corticospinal tract; CCing, cingulum bundle (cingulate gyrus portion); CHippo, cingulum bundle (hippocampal portion); CC, corpus callosum; IFOF, inferior fronto-occipital fasciculus; ILF, inferior longitudinal fasciculus; SLF, superior longitudinal fasciculus; UF, uncinate fasciculus; AF, arcuate fasciculus; L, left; R, right.

Supplementary Table S5. Group comparisons of tract-mean mean diffusivity (MD) in 20 major white-matter tracts derived from automated fiber quantification (AFQ).

Tract	n_pre:n_post:n_HC	Pre-CP	Post-CP	HC	ANOVA		Post-hoc groupwise (LSD)		
		(N=43)	(N=25)	(N=34)	F	p	Pre vs post	Pre vs HC	Post vs HC
ATR_L	40:24:33	0.815±0.060	0.779±0.061	0.777±0.033	4.791	0.011*	0.064	0.004**	0.938
ATR_R	39:23:34	0.795±0.076	0.801±0.049	0.791±0.052	0.065	0.938	0.825	0.831	0.734
CST_L	42:25:34	0.804±0.051	0.821±0.052	0.772±0.020	8.099	<0.001***	0.243	0.002**	0.001***
CST_R	39:24:32	0.783±0.057	0.828±0.057	0.785±0.027	3.201	0.047*	0.017*	0.852	0.026*
CCing_L	38:22:30	0.796±0.062	0.804±0.042	0.758±0.032	5.842	0.005**	0.691	0.003**	0.020*
CCing_R	38:22:30	0.780±0.069	0.775±0.033	0.750±0.033	1.465	0.242	0.898	0.097	0.480
CHippo_L	23:14:10	0.873±0.070	0.902±0.102	0.857±0.034	0.622	0.543	0.435	0.551	0.275
CHippo_R	22:14:8	0.870±0.074	0.936±0.001	0.836±0.032	1.321	0.284	0.342	0.245	0.173
CC Splenium	39:21:31	0.941±0.099	0.910±0.115	0.920±0.077	0.030	0.970	0.811	>0.999	0.811
CC Genu	38:20:34	0.884±0.075	0.884±0.074	0.847±0.033	3.496	0.035*	0.994	0.014*	0.125
IFOF_L	40:23:34	0.884±0.065	0.896±0.091	0.856±0.042	2.327	0.106	0.595	0.094	0.082
IFOF_R	41:22:33	0.871±0.066	0.900±0.071	0.846±0.037	3.051	0.054	0.258	0.080	0.036*
ILF_L	39:23:33	0.905±0.067	0.957±0.119	0.853±0.037	10.380	<0.001***	0.044*	0.004**	<0.001***
ILF_R	40:23:34	0.908±0.080	0.879±0.062	0.841±0.031	9.979	<0.001***	0.217	<0.001***	0.108
SLF_L	39:23:33	0.772±0.060	0.772±0.045	0.729±0.027	7.795	<0.001***	0.992	<0.001***	0.013*
SLF_R	39:23:33	0.769±0.066	0.789±0.056	0.739±0.027	4.410	0.016*	0.327	0.023*	0.014*
UF_L	39:22:34	0.857±0.044	0.837±0.098	0.829±0.031	2.093	0.132	0.326	0.046*	0.679
UF_R	36:23:34	0.847±0.055	0.876±0.074	0.825±0.036	3.963	0.023*	0.125	0.087	0.010**
AF_L	38:22:34	0.793±0.083	0.782±0.057	0.745±0.030	4.851	0.011*	0.638	0.003**	0.112
AF_R	38:22:31	0.763±0.038	0.799±0.078	0.749±0.026	4.321	0.018*	0.043*	0.221	0.005**

Note: Values are mean ± SD. n_pre:n_post:n_HC indicates the number of participants with valid tract reconstructions in the Pre-CP, Post-CP, and healthy control groups. One-way ANOVA across groups with Fisher's LSD post-hoc tests. *, **, and *** denote $p < 0.05$, $p < 0.01$, and $p < 0.001$, respectively. Diffusivity values are reported in 10^-3 mm^2/s. **Abbreviations:** ATR, anterior thalamic radiation; CST, corticospinal tract; CCing, cingulum bundle (cingulate gyrus portion); CHippo, cingulum bundle (hippocampal portion); CC, corpus callosum; IFOF, inferior fronto-occipital fasciculus; ILF, inferior longitudinal fasciculus; SLF, superior longitudinal fasciculus; UF, uncinate fasciculus; AF, arcuate fasciculus; L, left; R, right.

Supplementary Table S6. Group comparisons of tract-mean axial diffusivity (AD) in 20 major white-matter tracts derived from automated fiber quantification (AFQ).

Tract	n_pre:n_post:n_HC	Pre-CP (N=43)	Post-CP (N=25)	HC (N=34)	ANOVA		Post-hoc groupwise (LSD)		
					F	p	Pre vs post	Pre vs HC	Post vs HC
ATR_L	40:24:33	1.231±0.075	1.207±0.074	1.187±0.041	3.831	0.026*	0.305	0.007**	0.420
ATR_R	40:24:34	1.205±0.091	1.209±0.080	1.193±0.042	0.225	0.799	0.900	0.550	0.641
CST_L	42:25:34	1.420±0.089	1.469±0.071	1.411±0.039	2.941	0.059	0.049*	0.595	0.019*
CST_R	39:24:32	1.400±0.071	1.430±0.059	1.403±0.046	0.691	0.505	0.251	0.869	0.309
CCing_L	39:23:33	1.223±0.081	1.224±0.055	1.217±0.054	1.293	0.285	0.971	0.123	0.244
CCing_R	38:23:30	1.159±0.080	1.208±0.017	1.151±0.050	2.071	0.142	0.292	0.069	0.872
CHippo_L	23:14:10	1.266±0.069	1.303±0.110	1.266±0.060	0.442	0.646	0.363	0.985	0.411
CHippo_R	21:14:8	1.244±0.082	1.322±0.010	1.217±0.058	0.861	0.435	0.345	0.446	0.228
CC Splenium	39:21:31	1.617±0.088	1.495±0.250	1.636±0.093	2.719	0.075	0.056	0.562	0.024*
CC Genu	39:21:34	1.422±0.085	1.430±0.077	1.437±0.046	0.349	0.706	0.782	0.406	0.815
IFOF_L	41:21:34	1.312±0.061	1.334±0.112	1.314±0.054	0.350	0.706	0.421	0.896	0.456
IFOF_R	41:22:33	1.296±0.076	1.343±0.067	1.302±0.051	1.294	0.281	0.114	0.744	0.159
ILF_L	39:21:33	1.306±0.071	1.356±0.037	1.261±0.065	8.786	<0.001***	0.047*	0.008**	<0.001***
ILF_R	40:23:34	1.295±0.096	1.259±0.046	1.220±0.047	8.726	<0.001***	0.201	<0.001***	0.162
SLF_L	39:22:33	1.119±0.070	1.149±0.081	1.082±0.050	5.151	0.008**	0.211	0.023*	0.005**
SLF_R	39:22:33	1.151±0.086	1.155±0.066	1.123±0.046	1.539	0.222	0.857	0.116	0.226
UF_L	40:22:34	1.255±0.055	1.228±0.093	1.242±0.041	0.792	0.458	0.238	0.400	0.525
UF_R	39:22:34	1.224±0.071	1.254±0.060	1.229±0.053	0.821	0.441	0.203	0.741	0.289
AF_L	39:22:33	1.187±0.069	1.227±0.069	1.171±0.039	3.025	0.047	0.090	0.567	0.041*
AF_R	39:22:33	1.183±0.061	1.125±0.090	1.173±0.033	2.369	0.102	0.087	0.537	0.033*

Note: Values are mean ± SD. n_pre:n_post:n_HC indicates the number of participants with valid tract reconstructions in the Pre-CP, Post-CP, and healthy control groups. One-way ANOVA across groups with Fisher's LSD post-hoc tests. *, **, and *** denote $p < 0.05$, $p < 0.01$, and $p < 0.001$, respectively. Diffusivity values are reported in 10^-3 mm^2/s. **Abbreviations:** ATR, anterior thalamic radiation; CST, corticospinal tract; CCing, cingulum bundle (cingulate gyrus portion); CHippo, cingulum bundle (hippocampal portion); CC, corpus callosum; IFOF, inferior fronto-occipital fasciculus; ILF, inferior longitudinal fasciculus; SLF, superior longitudinal fasciculus; UF, uncinate fasciculus; AF, arcuate fasciculus; L, left; R, right.

Supplementary Table S7. Group comparisons of tract-mean radial diffusivity (RD) in 20 major white-matter tracts derived from automated fiber quantification (AFQ).

Tract	n_pre:n_post:n_HC	Pre-CP (N=43)	Post-CP (N=25)	HC (N=34)	ANOVA F	ANOVA p	Post-hoc groupwise (LSD) Pre vs post	Pre vs HC	Post vs HC
ATR_L	39:23:33	0.609±0.072	0.565±0.065	0.572±0.038	3.773	0.028*	0.053	0.016*	0.741
ATR_R	38:22:30	0.589±0.082	0.597±0.054	0.590±0.067	0.028	0.973	0.817	0.985	0.828
CST_L	38:25:34	0.497±0.077	0.498±0.075	0.453±0.030	4.946	0.002**	0.969	0.005**	0.037*
CST_R	38:23:28	0.478±0.076	0.528±0.097	0.478±0.041	2.093	0.131	0.060	0.923	0.057
CCing_L	37:23:32	0.584±0.080	0.594±0.063	0.528±0.045	6.797	0.010**	0.709	0.001**	0.014*
CCing_R	38:23:29	0.589±0.073	0.558±0.044	0.549±0.049	2.068	0.139	0.442	0.052	0.818
CHippo_L	23:24:10	0.681±0.084	0.702±0.097	0.652±0.038	0.727	0.491	0.642	0.331	0.296
CHippo_R	20:14:9	0.691±0.072	0.744±0.001	0.646±0.027	1.919	0.167	0.439	0.103	0.166
CC Splenium	20:21:31	0.609±0.115	0.618±0.070	0.563±0.074	1.768	0.181	0.865	0.091	0.274
CC Genu	35:21:34	0.620±0.099	0.611±0.091	0.552±0.036	6.758	0.002**	0.788	<0.001***	0.058
IFOF_L	39:22:34	0.673±0.074	0.678±0.090	0.627±0.046	4.387	0.016*	0.868	0.010*	0.044*
IFOF_R	35:22:34	0.664±0.069	0.679±0.074	0.619±0.040	5.899	0.004**	0.590	0.003**	0.026*
ILF_L	39:21:33	0.706±0.070	0.750±0.118	0.650±0.038	10.080	<0.001***	0.093	0.002**	<0.001***
ILF_R	34:22:34	0.718±0.078	0.688±0.075	0.651±0.034	9.671	<0.001***	0.210	<0.001***	0.118
SLF_L	37:23:33	0.600±0.068	0.584±0.051	0.553±0.029	6.485	0.003**	0.404	<0.001***	0.111
SLF_R	37:22:33	0.582±0.065	0.605±0.070	0.548±0.035	5.158	0.008**	0.264	0.016*	0.008**
UF_L	39:22:34	0.657±0.062	0.614±0.104	0.623±0.041	2.136	0.126	0.530	0.044*	0.424
UF_R	36:22:34	0.667±0.068	0.687±0.078	0.623±0.038	6.676	0.002**	0.378	0.003**	0.005**
AF_L	36:22:33	0.576±0.089	0.559±0.068	0.532±0.039	3.046	0.054	0.539	0.017*	0.294
AF_R	34:22:31	0.563±0.057	0.586±0.079	0.537±0.037	3.208	0.048*	0.299	0.077	0.030*

Note: Values are mean ± SD. n_pre:n_post:n_HC indicates the number of participants with valid tract reconstructions in the Pre-CP, Post-CP, and healthy control groups. One-way ANOVA across groups with Fisher's LSD post-hoc tests. *, **, and *** denote $p < 0.05$, $p < 0.01$, and $p < 0.001$, respectively. Diffusivity values are reported in 10^-3 mm^2/s. **Abbreviations:** ATR, anterior thalamic radiation; CST, corticospinal tract; CCing, cingulum bundle (cingulate gyrus portion); CHippo, cingulum bundle (hippocampal portion); CC, corpus callosum; IFOF, inferior fronto-occipital fasciculus; ILF, inferior longitudinal fasciculus; SLF, superior longitudinal fasciculus; UF, uncinate fasciculus; AF, arcuate fasciculus; L, left; R, right.
